# Supplementary material for: Are carbon emissions trading and green financial instruments synergistic? -Comprehensive quantitative research based on content analysis
Source: PLoS One. 2024 Mar 7;19(3):e0298601. doi: 10.1371/journal.pone.0298601 (PMC10919601; doi:10.1371/journal.pone.0298601)
Supplement: S1 Data — (ZIP) [file pone.0298601.s001.zip › Data/地级市Z_F工作报告.zip!/╣π╢1⁄2/╓Θ║ú╩╨/└┤╫╘╣1⁄2╓┌║┼í╛┬φ┐╦╩2╛▌═°í┐_2019.docx]

各位代表：

　　我代表珠海市人民政府，向大会作政府工作报告，请予审议，并请政协委员和其他列席人员提出意见。

　　一、2018年工作回顾

　　2018年是珠海改革发展史上具有重要意义的一年。10月22日，习近平总书记亲临广东视察，第一站便来到珠海，考察了横琴粤澳合作中医药科技产业园和格力电器公司；10月23日，总书记出席港珠澳大桥开通仪式并宣布大桥正式开通；11月6日，总书记致信祝贺第十二届中国国际航空航天博览会开幕。总书记对珠海工作的高度重视和亲切关怀令全市上下备受鼓舞、倍感振奋，倍增新时代改革开放再出发的信心和力量。一年来，在省委、省政府和市委的坚强领导下，在市人大、市政协的监督支持下，我们全面贯彻落实习近平新时代中国特色社会主义思想和党的十九大精神，学习贯彻习近平总书记对广东重要讲话精神，认真落实省委十二届四次、五次全会和市委八届五次全会精神，坚持稳中求进工作总基调，贯彻新发展理念，落实高质量发展要求，统筹做好稳增长、促改革、调结构、惠民生、防风险各项工作，有效应对外部环境变化，扎实推动珠海经济特区“二次创业”，经济社会发展取得新成效。

　　2018年政府工作报告部署的8个方面159项具体工作，149项已经完成，10项根据发展情况变化做了调整。十件民生实事包含的22项具体任务，21项已经完成，1项基本完成。

　　初步统计，全市实现地区生产总值2966亿元、增长8%；固定资产投资1858.57亿元、增长20.7%；社会消费品零售总额1162亿元、增长7.6%；进出口总额3246.28亿元、增长8.5%；一般公共预算收入331.47亿元、增长5.4%；全体居民人均可支配收入48007元、增长9%；居民消费价格涨幅为2.3%；城镇登记失业率为2.25%。

　　（一）构建高质量发展体制机制开局良好

　　“破、立、降”取得积极成效。破除无效供给，基本实现“僵尸企业”市场出清，全面完成省下达淘汰落后产能任务。加快培育新动能，全市新增商事主体7.1万户，其中企业3.9万户，先进制造业、装备制造业、高技术制造业增加值占规模以上工业比重分别达54.8%、39%、29.3%。落实减税降费政策，累计为企业减负262亿元。促进土地节约集约利用，出台完善工业用地供应、加强项目用地开竣工监管等政策，新增建设用地702公顷，收储土地186公顷，处置闲置用地622公顷。

　　营商环境持续优化。实施营商环境综合改革行动方案，出台全省首部公共服务地方标准管理办法，企业开办时间和社会投资项目审批时间进一步缩减，政府公共服务总体满意度全省排名第三。深入推进“放管服”改革，下放市级事权108项，公布市级行政审批中介服务事项117项，取消证明事项134项。优化“一门式一网式”服务，348项办理事项实现“最多跑一次”，1302个政务服务事项实现“全城通办”。完成商事主体登记“多证合一”改革。国家信用示范市创建通过初评。

　　各类市场主体共同发展。完善国企改革发展政策体系，推动国有经济提质增效，市属国有资产总额达7183.68亿元、所有者权益达2595.33亿元、归属国有权益达1181.77亿元，分别增长16.8%、28.9%、19.3%。落实省“民营经济十条”，新增2个国家中小企业公共服务示范平台、3个省级小微企业双创示范基地，新设民营企业3.2万户、增长36.3%，民营经济固定资产投资额达864.07亿元、增长27.3%。落实省、市“外资新十条”，在市场准入、用地保障等方面优化政策，新设外资企业3973户、增长153.9%，实际吸收外资156.3亿元、增长26.9%。

　　（二）现代化经济体系建设扎实推进

　　实体经济发展态势较好。落实省、市“实体经济新十条”，在促进工业投资、技术改造、装备制造、智能制造等方面出台系列政策。投入109.7亿元完善园区基础设施，实施“十百千企业腾飞行动”，规模以上工业增加值达1082.14亿元、增长14%，工业对经济增长的贡献率达76.1%。与华为、富士康、华大基因、正大集团、平安集团等行业龙头开展战略合作，新引进好迪、优必选等77个亿元以上项目，伟创力工业4.0旗舰园、中兴智能汽车等128个亿元以上项目加快建设，福陆海工、恩捷新材料等49个亿元以上项目建成投产。加快创建省军民融合创新示范区，鲲龙AG600飞机水上首飞成功，云洲智能中国首艘导弹无人艇成功试射，无人机验证飞行空域获批设立。

　　创新驱动战略深入实施。落实科技创新三年行动计划，出台培育引进“独角兽”企业、创新产品首购订购等政策，全社会研发经费支出占地区生产总值比重达2.8％，每万人口发明专利拥有量64件、有效注册商标拥有量352件。格力电器等企业获得第20届中国专利金奖5项、占全省一半。扎实推进创新平台载体建设，抓住省启动建设第二批实验室的机遇，争取到南方海洋科学与工程省实验室在珠海布局，新增2家国家级、17家省级技术中心、56家省级工程技术研究中心和2家省级制造业创新中心，复旦创新研究院、横琴科学城加快建设，华南理工创新研究院、智慧产业园先导区投入使用，国家耗材、船舶及海工质检中心服务能力不断提升。规模以上工业企业研发机构覆盖率达41%。成功举办中国集成电路设计业年会。创新主体蓬勃发展，高新技术企业总数突破1900家，欧比特成功发射5颗高光谱卫星，纳睿达研发出相控阵雷达超精细化天气观测系统，光驭科技获中国创新创业大赛新材料行业成长组一等奖。

　　金融和人才支撑作用不断加强。出台实施“金融十四条”，建立风险补偿和政府金融资源激励机制，综合施策支持普惠金融发展，着力缓解企业融资难融资贵问题，中小微企业通过“政企云”投融资增信子平台获得贷款101.35亿元。出台科技融资奖励和科技信贷、创业投资风险补偿等措施，设立粤港澳前沿产业投资基金、科技天使基金。外商投资股权投资企业试点落地实施，跨境投融资便利化取得实质进展。抢占金融科技产业发展先机，市金融科技中心加快建设，横琴智慧金融产业园正式运营。出台珠海英才计划，举办系列人才主题活动，配建人才住房1171套，引进各类人才2.7万名、增长50%。5人入选国家创新人才推进计划，新增博士后工作站6家、博士工作站24家。

　　（三）全面开放新格局加快形成

　　自贸区制度创新提速。新推出18个方面66项改革创新举措，横琴对优质发展要素的吸引力进一步增强，新落户2家世界500强、1家中国500强企业，首届科技创业大赛吸引全球952家优秀科创团队参赛。一批制度创新成果领先全国，首创税务诚信报告免责体系，首个互联网金融仲裁平台投入运行，率先实施自贸区供用电规则，主导制定综合管廊运维管理国际标准，远程可视自助办税平台案例获国务院办公厅通报表扬，国际互联网数据专用通道获批，首家内地与港澳三地联营设计顾问机构挂牌运作。

　　区域合作深入推进。港珠澳大桥及口岸正式开通，“合作查验、一次放行”新模式落地实施。新签订金融、投资贸易、青年实习等珠澳合作协议，新成立珠澳青年交流促进会、珠港澳物流联盟，新增珠港、珠澳姊妹学校15所。成功举办粤港澳大湾区妇女创新创业大赛、首届“粤菜师傅”技能大赛。在全省率先为港澳台同胞提供住房公积金服务。第四条对澳供水管道基本完工，对澳供电全电缆线路通道投入使用。横琴粤港澳紧密合作示范区加快建设，新注册港澳企业774家、总数达2744家。粤澳合作产业园5平方公里净用地明确划定、21个项目开工。粤澳合作中医药科技产业园累计注册企业111家、孵化区科研总部大楼等投入使用，澳门青年创业谷新引进港澳项目47个、累计孵化港澳项目181个，跨境说网络科技等创新型企业快速成长。澳门单牌机动车入出横琴配额达2500辆。启用琴澳跨境视频调解平台，跨境消费维权实现“零跑动”。万山区与澳门共建的海洋功能食品研发中心、珊瑚研究中心、海洋海岛教学研究基地加快推进。实施“珠台52条”，台胞在珠发展更加便利。

　　开放水平不断提升。出台实施稳外贸系列政策，新增2家国家级外贸转型升级基地，获国务院批准建设跨境电商综合试验区。外贸结构持续优化，高新技术产品进出口增长24.5%，对“一带一路”沿线国家地区进出口增长8.3%。城市对外交往影响力进一步提升，成功举办全球服务外包大会、中德人工智能大会、中以科技创新投资大会、中拉企业家高峰会、央视春晚分会场、中国国际马戏节、“海丝”论坛、亚洲帆板和风筝板锦标赛等活动。第十二届航展获得社会各界的广泛好评，43个国家和地区的770家厂商参展，现场签约合同金额212亿美元。新增5个国际友好（交流）城市，总数达32个、居全省地级市首位。加快创建国家全域旅游示范区，共接待游客4311.31万人次、增长8.3%，实现旅游总收入466.16亿元、增长26.8%。

　　（四）营造共建共治共享社会治理格局取得实效

　　底线保障水平持续提升。九项民生支出399.93亿元、增长12.9%，占一般公共预算支出的69.8%。出台“促进就业十条”，新增城镇就业4.7万人。基本养老保险基础养老金由每人每月380元提高至400元。城乡低保、特困人员、户籍孤儿基本生活标准分别由每人每月896、1434、1580元提高至980、1568、1710元。基本医疗保险补贴由每人每年510元提高至550元，基本医疗保险家庭病床支付比例由70%提高至80%。人均生育津贴达1.2万元。为全市户籍残疾人购买每人100元的意外伤害保险，贫困重度残疾人集中托养补贴标准增幅达40%。精准帮扶困难职工标准由每人每年3000元提高至6000元。建成1140套保障性住房、3818套棚户区改造住房。公积金缴存覆盖率保持全省第一。

　　公共服务体系不断完善。全市新增普惠性幼儿园学位3838个、义务教育公办学位7860个，梅华幼儿园、格力小学、横琴一中、金湾一中、市技师学院金湾校区等投入使用，华中师大珠海附中签约落地。高等教育加快发展，在校研究生、引进院士数量增长均超过20%。成功举办第四届中国教育创新成果公益博览会。新增医疗机构126家、健康细胞单元127个，家庭医生签约服务重点人群签约率超过70%。新建国家级胸痛中心、国医大师工作室等高水平诊疗中心，市人民医院、中大五院获评粤港澳大湾区高水平医院50强，省中医院珠海医院新住院大楼投入使用，市妇幼保健院异地新建项目主体结构封顶。20个社区体育公园建成使用，13个文化站升级改造，28个市民艺术中心加快建设。市图书馆及金湾、斗门图书馆获评国家一级馆。香湾、白藤等一批街道养老机构投入使用。新建45公里市政燃气管道，完成8.1万户老旧小区户外公共管道加建工作。供电可靠性排名全省第一。

　　社会治理能力得到加强。支持社会组织健康有序发展，全市依法登记社会组织达2422家、民办社工机构达82家，注册志愿服务组织达2248个、志愿者达35.3万人。启动“城市社会治理基础单元”改革试点，建立健全“十户联保、小组联防、村村联动”的农村基层治理新机制，数字城管延伸覆盖至所有村落，荣获全国社会治理创新示范市，香洲区成为全国社会治理和服务创新实验区。全面集中市级部门行政复议权，行政争议有效化解。全省食品安全示范城市创建通过中期评估。安全生产形势持续稳定，事故死亡人数下降10.9%，没有发生较大以上生产安全事故。强化自然灾害风险防控，建成一批海堤河堤加固达标工程，完成35处地质灾害隐患点治理。深入推进扫黑除恶专项斗争，打掉涉黑团伙4个、恶势力犯罪集团9个，查处“保护伞”38人。深化社会治安防控体系建设，发布“平安指数”2.0版，接报违法犯罪警情、刑事立案、严重暴力犯罪案件分别下降14.5%、4.9%和20.7%。圆满完成港珠澳大桥通车仪式、航展等重大活动安全保卫工作。

　　（五）城市建设步伐加快

　　交通建设加速推进。推动珠海机场升级改造，航站楼保障能力翻番，旅客吞吐量达1122.1万人次、增长21.7%。莲洲通用机场一期顺利完工。黄茅海5万吨级航道一期工程开工，高栏港区集装箱码头二期、东澳岛客货运码头等项目建设进展顺利，珠海港货物吞吐量达1.38亿吨。洪湾中心渔港开港。高铁通达城市达52个，乘坐高铁和城轨出行人数达2315万人次、增长29%。新开工板樟山隧道扩建、兴业快线南段、珠机城轨二期三个交通骨干工程，香海大桥、洪鹤大桥、鹤港高速一期、金琴快线、兴业快线北段等项目加快建设，珠机城轨一期全线贯通。完成88项道路挖潜，打通14条断头路，新建5座人行天桥，实现127处信号灯智慧联网控制。出台公交优先新措施，新开通826路特快专线等公交线路10条，优化线路371条，建成公交专用道6条，更新投放新能源公交车1153台，票价调至“全城1元”，公交日均载客量突破100万人次。加快完善陆岛交通，新开4条航线，固定航班由5个增加到19个，新投放4艘高速客轮，桂山岛开通夜航，往来海岛更为便捷舒适。

　　新区新城建设有序铺开。出台横琴、保税区、洪湾片区一体化发展规划，十字门隧道、南湾大道改造工程动工，横琴隧道建成通车，瑞吉酒店、乾元酒店开业。西部生态新城建设提速提质，金湾片区公共文化中心完工，商务中心、服务中心和艺术中心加快建设，斗门片区大信新都汇开业运营，富山新城邻里中心开工，平沙新城生活配套加快完善。唐家科教新城建设持续推进，悦椿酒店开业，前环片区配套市政道路基本建成。

　　城市面貌明显提升。着力营造干净、整洁、安全、优美的城市环境，顺利通过全国卫生城市复审，恢复全国文明城市荣誉称号。启动文明珠海“随手拍”，认真办好量大面广的群众身边“小事”。开展泥头车专项整治，推动余泥渣土密闭运输，统一规范建筑工地围挡，推动主城区商铺“入室经营”，基本完成户外大型立柱广告清拆。超额完成省下达的违法建设整治任务。61条道路完成路面改造美化，主干道保洁水平显著提高。香山湖公园、海天驿站、大门口湿地公园等建成开放，情侣路、迎宾南路、前山立交、机场东路、城市部分出入口等重点区域环境提升成效明显。新建60公里绿道、70公里林荫道、40公里健康步道。完成香山古驿道6公里精华段修复，以古道串起古镇古村，让更多人品味香山古韵。新增三旧改造258公顷、完成改造201公顷，实施老旧小区微改造60公顷，启动上冲、东桥、翠微等旧村改造，兴业玻璃厂、恒信工业城等21个旧厂房改造项目全面推进。

　　（六）三大攻坚战成效初显

　　重点领域风险有效防控。加强政府债务管理，全面梳理处置政府和社会资本合作项目，政府债务保持在合理水平。超进度完成非法集资陈案处置工作，互联网金融合规性整改工作扎实推进，涉众型金融风险有效化解。坚持“房子是用来住的，不是用来炒的”定位，房地产市场调控政策取得阶段性预期效果，商品房销售价格保持基本平稳。持续推进国有企业降杠杆，企业的经营水平和风险防控能力得到提高。

　　污染防治攻坚战取得成效。推进中央环保督察“回头看”16个问题整改，完成全国集中式饮用水水源地环境保护专项行动23个问题整改。新增污水管网190公里，新增污水处理能力10万吨/天，富山工业园第一水质净化厂动工建设，全市地表水国考、省考断面水质100%达标，饮用水源水质100%达标，12条黑臭水体基本实现年度治理目标。全国水生态文明城市建设试点通过验收。全面开展建筑工地“六个100%”扬尘防控，推进道路扬尘、工业废气污染治理，传统能源公交车全部淘汰，陶瓷行业生产线“煤改气”全部完成，PM2.5年均浓度为27微克/立方米、比上年下降10%，空气质量在全国169个重点城市排名第11位。农用地、重点行业企业用地土壤污染状况详查扎实推进。

　　乡村振兴和脱贫攻坚有力推进。推动农业高质量发展，新增国家级示范合作社1家、省级特色小镇2个、省级农业龙头企业3家、省名牌农产品16个，莲洲镇被评为“中国禾虫之乡”，白蕉镇昭信村成功创建全国一村一品示范村，全国第一艘半潜船形智能化养殖渔场顺利投放，广东（珠海）种业博览会成功举办。实施村庄整治规划编制指引，改善农村人居环境，推进“三清三拆三整治”，清理积存垃圾14万立方米，建成垃圾收集点1106个，完成标准化公厕改造148座，拆除危旧房1866间，改善低收入群体住房553户。认真落实中央和省交办的扶贫任务，实施对口怒江产业帮扶项目45个、转移就业6439人，对口支援藏区库区项目31个，对口阳江、茂名精准扶贫产业项目609个。对口帮扶阳江的24个亿元以上项目落地建设。

　　一年来，我们按照省委和市委的决策部署，深入开展“大学习、深调研、真落实”工作，推动“思想大解放、作风大转变、效率大提升”，坚决全面彻底肃清李嘉、万庆良恶劣影响，持续深入推进中央巡视、中央扫黑除恶督导、国务院大督查等反馈问题整改，加强审计结果运用，强化廉政风险防控，建设法治政府，在全省依法行政考核中获评优秀，政府自身建设进一步加强。提请市人大审议法规草案5部、制定政府规章3部，认真办理市人大建议119件、市政协提案375件，顺利办结职业教育、交通建设等议案。双拥、民族、宗教、侨务、人防、气象、档案、流渔、供销、地方志、政务公开、海防打私、国防动员等工作取得新成效。

　　各位代表，2018年9月，强台风“山竹”来袭，创下珠海最大10分钟平均风力记录。面对大风、大浪、大潮叠加，我们科学部署、积极应对，及时转移10.8万名群众，果断实施交通管制，与澳门联动关闭所有口岸，在全体市民的积极配合下，做到“人不出门、车不上路”，实现了重大灾情面前无重大伤亡、无重大险情的防控目标。

　　各位代表，过去一年的成绩来之不易，这是党中央国务院、省委省政府和市委正确领导的结果，是上级各部门大力支持的结果，是全市人民共同奋斗的结果。在此，我代表市政府，向一直以来为珠海建设发展付出辛勤劳动的广大市民，表示崇高的敬意！向给予政府工作大力支持的人大代表、政协委员、各民主党派、各人民团体、各界人士，向关心支持珠海发展的中央驻珠单位、驻珠部队、省直部门、港澳台同胞、海外侨胞和国际友人表示衷心的感谢！

　　各位代表，我们也清醒地看到，政府工作还存在不少问题和不足。一是高质量发展存在不少短板。实体经济发展基础薄弱，拥有核心技术的创新企业不多。园区布局碎片化、同质化，产业集聚度不高。服务业发展与珠江口西岸核心城市的定位不匹配，占比低于全国和全省平均水平，商业氛围不浓、人气不旺、消费规模偏小。生态环境在相当程度上依赖自然禀赋，污水厂网存在历史欠账，固体废物处理能力有待提高。教育、医疗、养老等优质公共服务供给不足，城市建设管理不够精细。二是交通不畅依然是制约城市发展的突出问题。近年来交通建设虽有所提速，但内畅外联的交通主骨架仍未形成。对外大通道不足，市内快速通道没有成网成环，现有交通体系难以承载大湾区建设日益增长的人流物流，公交出行分担率较低，上下班高峰期拥堵问题仍未根本改善。三是统筹城乡、陆岛协调发展的力度仍需加大。乡村海岛发展与城区相比仍有较大差距，农村人居环境亟需全域整治提升，海岛基础设施、陆岛交通建设亟需加快。都市现代农业和海洋经济的发展水平，与珠海毗邻港澳的区位优势和拥有的丰富海洋海岛资源不相称，优质农产品、乡村及海岛旅游的品牌效应还不明显。四是对澳合作有待深化。横琴自贸区建设的活力与内涵还需进一步提升，公共服务配套尚不完备，产城融合需要提速，对澳门居民和投资者的吸引力有待增强，亟需推动更多创造型、引领型改革，在促进澳门产业多元发展上发挥更大作用。五是营商环境需进一步优化。创业就业及发展机会不充分，人才吸附能力不强，汇聚发展要素能力有限。民营企业及中小企业在用地用电、用工用料、市场开拓、融资等方面仍然存在不少困难，需要我们在加强招商引资工作的同时，更加注重对现有企业的帮扶和服务。对这些问题，要勇于面对，采取有效措施，切实加快解决。

　　二、2019年工作部署

　　2019年是中华人民共和国成立70周年和全面建成小康社会关键之年，是澳门回归20周年、珠海建市40周年。习近平总书记对广东重要讲话和对珠海工作重要指示，为珠海经济特区新时代改革开放再出发指明了前进方向、提供了根本遵循。我们要深刻体会习近平总书记对珠海的信任与重托，牢牢把握粤港澳大湾区建设和港珠澳大桥通车带来的历史性机遇，积极应对国内外形势变化，进一步增强机遇意识、忧患意识、进取意识，自觉把珠海的工作放在中央和省赋予的战略定位中、放在全国全省发展的大格局中，切实肩负起新时代的新使命，推动思想再解放、改革再深入、工作再落实。要更加注重把握好促进澳门产业多元发展和加快自身发展的关系、城市能级量级提升和生态环境保护的关系、推动制造业高质量发展和加快现代服务业发展的关系、提升城市品质和招商引资的关系、集中财力办大事和激发区级发展活力的关系、谋大事和抓小事的关系，既对标最高最好最优、又落细落小落具体，真正把发展机遇转化为发展优势，加快建设新珠海、新经济、新生活，打造粤港澳大湾区经济新引擎、独具特色令人向往的大湾区魅力之城和践行新发展理念的典范城市，为全国改革开放探索新鲜经验，为促进港澳融入国家发展大局、维护港澳长期繁荣稳定贡献力量，努力在广东构建“一核一带一区”区域发展新格局、实现“四个走在全国前列”、当好“两个重要窗口”中发挥更大作用。

　　做好今年政府工作的总体要求是：高举习近平新时代中国特色社会主义思想伟大旗帜，全面贯彻党的十九大和十九届二中、三中全会以及中央经济工作会议精神，认真贯彻落实习近平总书记对广东重要讲话和重要指示批示精神、在庆祝改革开放40周年大会上的重要讲话精神，全面落实省委十二届五次、六次全会和市委八届五次、六次全会部署，统筹推进“五位一体”总体布局，协调推进“四个全面”战略布局，坚持稳中求进工作总基调，坚持以人民为中心的发展思想，坚持新发展理念，坚持推动高质量发展，坚持以供给侧结构性改革为主线，坚持深化市场化改革、扩大高水平开放，统筹推进稳增长、促改革、调结构、惠民生、防风险工作，打好三大攻坚战，着力提高发展平衡性和协调性，坚持不懈推进全面从严治党，深入落实“1+1+9”工作部署，保持经济持续健康发展和社会大局稳定，以优异成绩庆祝中华人民共和国成立70周年。

　　建议2019年经济社会发展的预期目标和约束性指标为：地区生产总值增长7.5%左右；规模以上工业增加值增长10%；固定资产投资增长10%；社会消费品零售总额增长8%；外贸进出口保持正增长；一般公共预算收入增长7%；居民人均可支配收入增长8%；居民消费价格涨幅、城镇登记失业率均控制在3%以内；城镇新增就业3万人；主要污染物总量减排和大气、水环境质量完成省下达的目标。

　　围绕上述发展目标，重点安排以下工作：

　　（一）全面深化改革开放

　　深度融入粤港澳大湾区建设。把粤港澳大湾区建设作为全面深化改革开放的大机遇、大文章抓紧抓实办好，坚持设施“硬联通”和机制“软联通”并举，促进各类要素便捷流动和优化配置，共建富有活力和国际竞争力的一流湾区和世界级城市群。管好用好港珠澳大桥，谋划建设广江珠澳高铁、广中珠澳高铁和伶仃洋公铁通道，更好承接港深、广佛极点的辐射，加快建设珠江口西岸核心城市，携手澳门打造大湾区经济新增长极。在口岸通关、双向投资、多元合作、贸易便利化等方面积极探索，推动规则相互衔接，构建新型合作模式。推动专业资格互认，落实技术移民政策，促进跨境交流交往、创新创业。主动对接“广州—深圳—香港—澳门”科技创新走廊建设，建立跨境产学研合作机制，探索在生物医药、海洋科技等领域共建特色科创中心。建设珠澳跨境金融合作示范区，扎实推进外商投资股权投资企业试点。建设珠港澳粤菜研究院。携手港澳发展港珠澳大桥旅游，推动粤港澳游艇自由行全面落地实施。加强公共服务政策对接，研究制定更多便利港澳居民在珠发展措施。开展港澳青年来珠实习计划、青年同心圆计划。联合澳门共建中国设计博物馆，推动香山古驿道与澳门历史街区步行系统对接。加快平岗至广昌原水供应保障工程和广南梅供水管道工程建设，建成对澳供电第三通道。携手港澳深度参与国际合作，共建“一带一路”重要支撑区，建设好中拉经贸合作园、中以科技创新产业园、中德人工智能研究院等平台，积极融入全球经贸创新网络，务实开展人文领域交流合作。

　　牢记初心推进横琴建设。深入贯彻落实习近平总书记视察横琴重要指示精神，提高政治站位，强化政治担当，牢牢把握横琴开发建设的正确方向，不辜负总书记的殷切嘱托，主动加强对澳合作，丰富合作内涵，拓展合作空间，发展新兴产业，促进澳门经济发展更具活力。担当好先行先试的重要职责，争取国家支持创新分线管理制度等标志性重大改革事项，打造粤澳特别合作区。全面梳理现有土地使用和项目建设情况，提高粤澳合作项目比重，重点发展中医药、高新技术、特色金融、会展、休闲旅游等产业。按照“澳门资源+全球技术+创新人才+横琴载体”模式，加快粤澳合作产业园、横琴澳门青年创业谷建设。争取先行先试医疗健康政策，推动粤澳合作中医药科技产业园加快发展。建设横琴金融岛，设立澳门特色金融服务基地。加快横琴科学城建设，携手澳门共同发展特色芯片设计、测试和检测等相关产业。推动横琴国际休闲旅游岛相关政策落地，配合澳门建设世界休闲旅游中心。实施“港资港模式、澳资澳模式”，创新工程审批管理。将澳门莲花口岸整体搬迁至横琴，实施“合作查验、一次放行”通关模式。制定跨境办公专项政策，鼓励澳门企业在横琴跨境办公。深化民生合作，争取开工建设集养老、居住、教育、医疗等功能于一体的综合民生项目“琴澳新街坊”。

　　推动改革举措落地生效。以落实机构改革为契机，加快政府职能转变，大幅减少政府对资源的直接配置，凡是市场能自主调节的就让市场来调节，凡是企业能干的就让企业干。继续推进“放管服”改革，尽可能把资源、服务、管理下沉到基层。深化供给侧结构性改革，落实好“巩固、增强、提升、畅通”八字方针。完善考核评估体系，推动园区差异化发展，促进产业集聚集群发展。做好第三次土地调查，强化项目用地监管，盘活低效用地，加大土地收储力度。开展亩产效益评价，促进正向激励和优胜劣汰，推动资源向优质企业和产品集中。持续深化营商环境综合改革，实施能评、环评等区域评估制度，推进审批标准化改革。落实“证照分离”“照后减证”，提高商事主体开办便利度。全面实施市场准入负面清单制度，破除各种隐性障碍，保障各类市场主体公平竞争。建立行政审批中介服务事项清单动态调整机制，促进中介服务市场健康发展。深化国资国企改革，减少管理层级和同质化竞争，提升国企竞争力。

　　（二）着力促进经济平稳增长

　　积极扩大有效需求。在先进制造业、现代服务业、现代化基础设施等领域谋划推进一批重大投资项目，安排年度重点项目208个、投资576亿元，力促36个项目建成投产、54个项目开工建设。在市场准入、用地保障、产权保护等方面优化投资环境，鼓励社会资本参与基础设施、旅游、养老等项目建设。积极开展促消费系列活动，加强市场监管，营造安全放心的消费环境。支持房地产合理自住需求，坚决遏制投机炒房。加快充电基础设施建设，落实好新能源汽车车辆购置税优惠政策，促进二手车便利交易，推动汽车消费优化升级。创建国家全域旅游示范区，提升旅游产业附加值。扩大文化产品供给，培育文化消费理念。促进信息消费，发展面向社区生活的线上线下融合服务。健全家政服务标准和服务规范，支持家政服务品牌建设。抓好外贸转型升级基地建设，新增1-2个外贸公共服务平台。促进服务贸易发展。培育外贸新业态，推进跨境电商“两平台六体系”建设。鼓励进口高新技术产品、先进设备和紧缺资源性产品。

　　实实在在解决企业困难。构建亲清新型政商关系，积极与企业接触沟通，主动热情做好服务，切实解决企业发展难题。畅通政企沟通渠道，健全企业家参与涉企政策制定机制，出台支持民营企业及中小企业高质量发展政策措施。继续降低企业用地用气用电成本，实施社会保险费率优惠政策。完善园区路网及生活配套设施，支持企业建设职工宿舍，实行重点企业技能型人才推荐入户，帮助企业留住员工。完善科技企业投融资和中小微企业信贷风险补偿机制，对企业实施重点项目建设给予贷款贴息支持，降低企业融资成本。发挥珠海基金作用，帮助有发展前景的企业纾解流动性困难。落实好自主创新产品首购订购政策，支持本地企业产品应用和市场推广。用好龙头企业专业服务资源池，带动中小企业发展提升。

　　防范化解重大风险。着力处置金融领域风险点，重点治理风险源头，加强金融风险排查预警，继续推进非法集资陈案化解，深入开展互联网金融领域风险整治。严格执行政府债务限额，坚决制止违法违规举债。深化预算编制执行监督管理改革，提升财政资源配置效率和使用效益。切实防范财政支出风险，树立过紧日子的思想，坚持有保有压，严控“三公”经费预算，把有限的财力用在刀刃上。建立房地产市场平稳健康发展的长效机制，保持调控政策连续性稳定性，把稳地价、稳房价、稳预期落到实处。着力化解国有企业经营和债务风险。

　　（三）加快建设现代产业体系

　　推动制造业高质量发展。做优产业存量，继续实施“十百千企业腾飞行动”，力争超百亿级企业达7家、高新技术企业达2100家。“一企一策”支持第一梯队企业发展，充分保障格力电器产业用地，配套建设伟创力小镇，支持丽珠集团等企业做大做强。推动联邦制药、光宇电池、晨新科技等第二梯队企业增资扩产，给予第三梯队科技型中小企业全生命周期扶持。实施新一轮工业企业技改三年行动计划，开展智能制造模式推广应用。做大产业增量，完善招商工作市级统筹机制，面向发达国家和国内大型企业开展精准招商，建立重大项目洽谈引进绿色通道，争取更多项目落地珠海。推进利盟激光打印机等68个亿元以上项目加快建设，推动烽火海洋网络设备等60个亿元以上项目建成投产。开展质量提升行动，构建与国际接轨的优质标准和认证体系，以品牌建设引领制造业提质增效。

　　大力发展战略性新兴产业。加快发展新一代信息技术、高端装备制造、生物医药、新能源及新能源汽车、节能环保、数字创意、新材料等产业，培育一批“独角兽”和“瞪羚”企业。推进软件产业转型升级，积极布局大数据、人工智能等产业，形成新一代信息技术产业集聚区。紧抓集成电路设计环节，集中力量引进集成电路全产业链项目，建设集成电路高端设计与制造基地。继续实施先进装备制造业三年行动计划。依托金湾生物医药产业基础、横琴粤澳合作医药发展平台，建设生物医药产业发展联动带。引导新能源及节能环保产业向西部集聚，建设绿色低碳产业示范基地。发展数字经济，建设窄带物联网、5G网络等信息基础设施，培育新兴数字产业应用，推动4K产业发展。高水平建设航空产业园、通用航空产业综合示范区，推动省军民融合“十三五”规划七项重点工程建设。

　　加快发展现代服务业。抓住港珠澳大桥开通给珠海服务业发展带来的绝佳机遇，积极吸引承接港澳优质服务业，汇集国内外高端服务业，提升商贸、旅游、会展、物流、金融等产业发展水平，争取服务业发展上台阶。精准引进国际顶级财团和实力企业，在港珠澳大桥珠澳口岸人工岛、高新区、西部生态新区等地建设一批商贸综合体和品牌酒店，满足大湾区优质生活圈消费需求。整合盘活闲置及低效使用的各类物业，着力发展楼宇经济，重点发展商务商贸、金融科技、文化创意等产业，落实加强总部企业发展实施办法，培育一批“专业楼”“亿元楼”。加快发展现代物流业，重点发展“珠港陆空联运”及拓展业务，推动第三方物流和冷链物流发展，建设电商快递结合产业园。依托珠海独特区位优势、一流会展场馆优势和已有展会品牌优势，引进和培育更多专业展会，做大做强会展经济。发挥生产性服务业在研发设计、流程优化、市场营销、物流配送、节能降耗等方面的引领带动作用，推进先进制造业与现代服务业深度融合。

　　增强自主创新能力和实力。发挥自贸区、自创区联动优势，力争在大湾区国际科技创新中心建设中占有一席之地。加强基础研究和应用基础研究，创新管理体制和运营机制，加快建设南方海洋科学与工程省实验室。发挥好空调设备及系统运行节能等国家重点实验室作用，推进电机与控制院士工作站、智慧海洋院士工作站、格力电器新一代人工智能开放平台等创新载体建设。支持企业建立研发机构，主动对接国家、省重大科技专项实施。深化与中山大学、澳门大学、复旦大学、暨南大学等合作，建设新型研发机构。完善企业孵化育成体系，力争科技企业孵化器达33家。建设标准国际化创新型城市、国家知识产权示范城市，筹建省海洋工程装备产业计量测试中心，加快建设新能源汽车国家质检中心，推动国家知识产权保护中心落地，建设好横琴国际知识产权交易中心。促进科技金融发展，引导横琴的私募股权基金、创业投资资本投资珠海企业。做好上市公司培育，鼓励企业抢滩科创板。落实好英才计划，深化人才评价和职称制度改革，开展特色引才活动，加大柔性引才力度，在子女就学、配偶就业、医疗保障等方面为人才提供高效便捷服务，出台人才安居管理办法，新建人才住房800套，力争全年引进各类人才4万名。

　　（四）开展新一轮交通大会战

　　加快构建交通主骨架。完善重点交通项目推进机制，做细做深前期工作，想方设法破解拆迁难题，着力推进交通骨干工程建设。推动对外通道畅通便捷。推进洪鹤大桥、鹤港高速建设，推动黄茅海大桥实施，构建港珠澳大桥直通粤西快速通道。加快兴业快线、金琴快线建设，有效连通深中通道。完善市内交通路网，启动金港大桥前期工作，加快金海大桥、香海大桥及支线建设，打造由金琴快线、香海支线、南湾大道、昌盛路、兴业快线、金唐东路构建的主城区环线。推动珠机城轨一期运营，争取把地铁纳入国家和省相关规划，启动城市轨道交通相关工作。

　　推进交通重要节点建设。尽快完成机场升级改造，推进航展停机坪租赁及改造、航空公司运营维修基地建设，补齐机场保障能力短板，支持开通更多航线。统筹好机场建设、航展中心提升、航空产业园发展，加快推进机场改扩建、机场综合交通枢纽、航空物流园建设。推动机场口岸对外开放，发挥珠港机场合作优势，拓展国际公务机业务。推进莲洲通用机场投入运营。开展港口规划修编，建设高栏港综合保税区，加快提升港口能级量级，完成黄茅海5万吨级航道一期工程，加快高栏港区集装箱码头二期工程建设，完成高栏港区15万吨级主航道工程竣工验收，推动对外开放水域进一步扩大。统筹推进口岸建设，完善口岸布局，加快九洲港口岸重建、湾仔口岸迁建、拱北口岸改造，完成横琴口岸旅检大厅、青茂口岸建设。

　　系统治理交通拥堵。启动珠海大桥扩建工程，实施珠海大道大货车高峰期限行。加快板樟山新增隧道建设，全面完成主城区道路沥青罩面改造。强化道路挖潜，打通断头路，建设一批人行天桥，优化完善交通信号灯和交通标识，提高道路通行能力。加强交通组织，保持路面见警率。强化科技治堵，推进智慧交通信号协调控制系统二期工程建设。加强“五类车”和交通违法行为整治，维护道路交通秩序。支持智能立体停车场建设。鼓励企业、园区、学校等开通通勤车。建设公交都市，加密公交线路，加大公交车投放，加强公交专用道执法，让公交出行更加便捷。

　　（五）提高发展平衡性和协调性

　　实施区域发展新战略。推进发展空间南进西拓北接东优，尽快拉开城市框架。南部对接港澳推进横琴、保税区、洪湾片区一体化建设，推进洪湾大道、十字门隧道、大横琴山隧道、横琴医院、横琴市民艺术中心等项目建设。加快西部生态新区建设，推进金湾创业大厦、斗门科创中心、富山产业服务中心、平沙新城中心公园、鸡啼门特大桥等项目进展，确保华中师大珠海附中首期建成招生。北部对接深中通道建设唐家科教新城，推进金琴快线北延线、高新互通立交建设，加快完善南北围、后环等片区路网。东部优化提升香洲主城区，加大城市更新推进力度，对老旧工业区和混杂在园区的城中村实施连片改造，为新兴产业发展和公共设施补短板腾出空间。加快“三溪”科创小镇、香洲科创中心、拱北口岸商业中心等项目进展，推动洪湾等旧村改造、凯威东大等旧厂房改造、夏湾农贸市场等旧城镇改造。加快“烂尾楼”盘活处置。

　　全面实施乡村振兴战略。落实乡村振兴战略规划，做强富民兴村产业，制定促进农村一二三产业融合发展、集体经济发展壮大等政策。做好良种及农业科技推广，建设高标准基本农田1.41万亩。推进国家农业科技园、台湾农民创业园、现代农业产业园建设，为大湾区居民供应优质农产品。实行点状供地政策支持乡村产业发展，加快岭南大地田园综合体、十里莲江、逸丰生态园等项目建设。实施农业龙头企业培优工程，支持农村电商发展。推进美丽宜居乡村建设，年内基本完成“三清三拆三整治”任务。围绕供水、供气、供电、水利、网络、道路、路灯、垃圾处理、污水治理、房屋改造等十大要素，推动农村基础设施提档升级。加强农村“三资”管理服务平台建设，完成农村集体产权制度改革。健全市内精准帮扶长效机制，继续加大对低收入群体的综合帮扶。扎实做好对口怒江扶贫协作、对口支援藏区库区和对口阳江、茂名精准扶贫工作，带动社会力量参与脱贫攻坚。拓展扶贫地区特色农产品销售渠道，丰富珠海市民菜篮子。做好对口阳江产业帮扶和对口黑河合作工作。

　　保护利用好海洋海岛资源。落实珠海经济特区海域海岛保护条例和无居民海岛开发利用管理规定，推动海洋海岛绿色可持续发展。加大对中华白海豚和珊瑚礁的保护。推进“美丽海岛”建设，加快渔村更新改造，建成大万山岛、东澳湾污水处理站，完成海岛存量垃圾转运整治。实施海岛基础设施建设三年投资计划，加快推进大万山岛环岛路、万山湾客运码头、桂山岛十三湾防波堤、大万山岛和外伶仃岛水库扩建等项目建设，加快陆岛危险货物运输码头、外伶仃岛石涌湾客货运码头防波堤、东澳岛和大万山岛客运码头防波堤前期工作。推动外伶仃岛大电网上岛，完善边远有居民海岛的通讯网络。引进国际知名企业，推动滨海旅游提质升级。建设省级海洋科创特色小镇。

　　（六）打造大湾区魅力之城

　　全面提升城市品质。坚持全市发展“一盘棋”，高标准编制国土空间整体规划，统筹各类空间性规划，推进“多规合一”和空间“一张图”管理，促进生产空间集约高效、生活空间宜居适度、生态空间山清水秀，成为粤港澳大湾区优质生活圈的重要组成部分。按照“山形可看可达可憩”“水体要清要动要养”“岸线自然安全品质”的要求，加强城市修补和生态修复。新建一座观光塔，打造十字门、九洲两个商务区，建设野狸岛、银坑半岛、石景山三大公共空间，擦亮港珠澳大桥、情侣路、万山群岛、横琴长隆四张名片，提升圆明新园、唐家古镇、斗门水乡、温泉酒店、特色商业街等五个片区的品质和内涵，打造独具特色的城市“打卡”目的地。实施景观亮化工程，新建大型建筑同步配建照明设施，完善地标建筑照明，打造璀璨雅致、动静相宜的都市夜景。以文化涵养城市品格，鼓励本土文艺创作，打造艺术之城、书香之城。新城新区要注重人文特色，对老旧街区更多采用微改造方式，修旧如旧、建新如故，在保留建筑肌理的基础上提升功能、传承文化。办好中国国际马戏节、莫扎特国际青少年音乐周、WTA超级精英赛、ATP250巡回赛、沙滩音乐节、留学生节、泛珠三角赛车节等活动。全方位加强城市宣传推介，展示更有韵味、更具活力的城市形象。

　　加强城市精细化管理。深化城市管理体制机制改革，整合部门职能，下沉工作资源，做实镇街网格化管理。健全城市管理法规体系，推进基础设施、园林绿化、地下空间等行业标准统一、相互衔接，制定洒水降尘、共享单车管理等政府规章，实现管理标准精细化。下足“绣花”功夫，对主干道、城市主要出入口、城轨站点、口岸、机场等周边环境进行综合提升。统筹规范供电、供水、电信、燃气、道路等各类标牌标识以及监控监测设施的建设设置。严控新栽大树、名贵树，合理移植现有树木，绿化美化更多通过撒种、育苗，营造花开花落、色彩丰富、四季更迭的自然生态景观，建设名副其实的花园城市。建立环卫保洁长效机制，提高环卫机械化水平。开展背街小巷专项整治，及时修复破损市政设施，不留卫生死角、不留管理盲区，确保每一个角落、每一寸土地都有人管理和服务。推进“厕所革命”新三年行动计划。用铁的手腕强化违法用地、违法建设整治。加强工地管理，杜绝施工扰民。支持有条件的老旧小区加装电梯。加快智慧城市应用项目建设，推进城市智能化管理。

　　巩固提升生态优势。推进“公园之城”和“千里绿廊”建设，新建20公里健康步道、40公里林荫道、9个乡村绿化美化点。建设百里碧道，打造绿色水域岸线空间。推进海绵城市试点建设，继续做好中欧低碳生态城市综合试点工作。建立水源保护区生态补偿逐年递增机制。全面落实河长制、湖长制，深化水环境综合治理，开展“五清”专项行动，力争完成17条黑臭水体整治。启动富山第二水质净化厂建设，加快井岸、白藤、新青等水质净化厂提标改造，完成香洲、拱北、吉大、南区等水质净化厂提标改造。深化排水管理体制改革，启动排水设施信息化管理系统建设，全面推进管网清淤和病害治理，新建改建污水管网610公里。推进土壤污染防治，重点做好农用地和建设用地土壤污染状况详查、分级分类管控、安全利用及治理修复工作。强化危险废物规范化处理处置监管，力争实现固体废物100%自主处理。加快推进中信生态环保产业园基础设施配套工程和环保生物质热电工程二期建设，开工建设污泥处置中心一期、医疗废物处置中心和餐厨垃圾处理一期工程。巩固提升空气质量，常态化落实工地管理“六个100%”，强化工业废气治理，持续整治“散乱污”企业，严惩偷排偷放，保住“珠海蓝”金字招牌。

　　（七）切实保障和改善民生

　　提高就业和社会保障水平。积极应对就业形势变化，完善就业创业扶持政策，制定公益性岗位管理办法，加大就业困难人员就业援助力度，有效促进在珠高校毕业生留珠就业创业，开展重点企业职业技能精准培训。落实工匠培育计划，大力弘扬工匠精神。实施“粤菜师傅”工程，建设5个培训基地。稳步提高社会保险待遇水平，基本养老保险基础养老金提高至每人每月430元，低保标准提高至每人每月不低于1020元。基本医疗保险补贴提高至每人每年590元。在全省率先实施附加补充医疗保险。探索建立工伤保险长期照护制度。加快推动市级养老服务机构及市社会福利中心二期建设。加大住房保障力度，拓宽住房保障覆盖面和保障形式，完善保障性住房管理平台建设，建立多主体供给、多渠道保障、租购并举的住房供应与保障体系。

　　大力发展教育卫生事业。推进基础教育资源均衡配置省级试点工作，出台西部地区教育振兴攻坚行动计划，落实第二期特殊教育提升计划、学前教育第三期行动计划。新增公办学校10所、改扩建学校12所，新增学位17320个。推进名师培养工程，试行合同聘任制教师管理模式。对接产业需求，优化职业教育专业结构，建设职业教育公共实训基地。办好中国教育创新成果公益博览会。建设高水平大学，推动中山大学珠海校区海洋科学等楼群建成使用、北师大珠海校区秋季招生。实施“健康珠海2030”规划，加强重点人群健康服务。深化公立医院改革，推进精准疾控。支持市人民医院、中大五院进军省高水平医院建设“登峰计划”，加快推进市人民医院科研综合楼及放疗中心、市妇幼保健院异地新建、市慢性病防治中心及配套道路建设，稳步推进市西部医疗中心建设。强化基层卫生服务，推动中医药适宜技术基层全覆盖，开展国家基层高血压医防融合试点工作。

　　健全公共文化服务体系。建立深化文明城市建设常态化机制，加强公民思想道德建设，持续提升市民文明素质。加快文化基础设施建设，推动市博物馆和规划展览馆对外开放，实现基层文化设施全部达标。完善全民健身公共设施，加强社区体育公园和小型足球场等建设。加快儿童活动场所建设，推动村居儿童之家全覆盖。保护传承好历史文化，做好革命历史文物、古镇古村和非物质文化遗产的保护利用，申报建设国家历史文化名城。

　　提升公共安全保障能力。深化安全生产领域改革，加强城市安全风险排查管控，坚决遏制较大以上生产安全事故发生。完善火灾防控体系，推进消防安全“三个十万”工程。建设市中心粮库和粮食批发市场，确保粮食安全。加强食品药品安全监管，开展餐饮服务街区整治。落实基础设施人防要求。实施海堤提升工程，开展市区水浸点和农村易涝区域综合整治。完善城乡社区治理，推进信访工作制度改革，健全社会矛盾纠纷多元化解机制。深化平安珠海建设，创建全国禁毒示范城市，构建多维一体的反恐工作新格局，升级加固社会治安防控体系。深入开展扫黑除恶专项斗争，铲除黑恶势力滋生土壤。严厉打击盗抢骗、黄赌毒及电信诈骗等新型违法犯罪。争创全国双拥模范城“九连冠”。加强智慧海防建设和打私工作。

　　扎实推进十件民生实事。一是提供更加便捷的公交服务。新增10条公交线路，重点开通跨区快速公交。新增投放500辆纯电动公交车。支持发展定制公交。增加微循环公交，打通园区、村居出行“最后一公里”。二是开展小学生校内课后服务。全市70%以上具备条件的小学为有需求的学生提供早餐、午餐及托管看护服务，有效解决家长的后顾之忧。三是建设长者饭堂。新建18处社区居家养老配餐试点，为老年人提供集中用午餐及助餐送餐服务，逐步解决老人做饭难、吃饭难的问题。推动有条件的长者饭堂拓展日间照料等服务。四是推进农贸市场改造。完成25家硬件设施残旧、不达标的农贸市场升级改造，为老百姓提供安全、整洁、卫生、舒适的购物环境。五是改善陆岛交通。完善海岛基础设施，推动陆岛交通客运公交化，进一步拓展航线、加密航班，市民享受8折船票优惠。六是加快普及管道天然气。新建40公里市政燃气管道，完成4万户老旧小区住宅的户外公共燃气管道加建工作。七是加强防灾减灾救灾能力建设。整治103处地质灾害隐患点。配强应急救援力量，充实抢险救灾物资和装备。八是完善基层派出所便民设施。修缮40个派出所，重建3个派出所。推进智慧派出所建设，推动窗口服务进驻各级行政服务中心。九是完善医疗卫生信息便民服务。在全市二级以上公立医院实施一码通用、一网联通、一键诊疗、一站会诊、一体服务的信息便民就医措施。十是提升园林管养水平。提升公园品质，做好主干道树木修剪和公共绿地日常管养，开工建设香炉湾城市阳台。

　　（八）加强政府自身建设

　　全面推进依法行政。强化法治思维，坚持科学民主依法决策，推行行政程序法定化，落实政府法律顾问制度，让改革发展各项工作始终沿着法治轨道前行。深化以区级为主的综合行政执法改革，推行行政执法“三项制度”。加强政务公开工作，及时回应社会关切。深入开展宪法宣传教育活动，加大全民普法力度。认真落实市人大及其常委会的各项决定，确保人大议案、建议和政协提案涉及问题得到有效解决。

　　提高行政服务效能。深化政务服务改革创新，打造数字政府，推动政务服务“全城通办”“一网通办”，拓展“粤省事”在珠海移动服务应用，建成数据互联互通公共资源交易平台。推动“一门式”综合受理模式向基层延伸。加强干部队伍建设，用好容错纠错机制，激励干部担当作为，开展好电视问政，强化行政执行力建设，让企业和群众真正感受到“思想大解放、作风大转变、效率大提升”的实际成效。

　　加强政府系统党的建设。落实新时代党的建设总要求，把坚持和加强党的全面领导贯彻到政府工作全过程、各领域。严明政治纪律和政治规矩，牢固树立“四个意识”、坚定“四个自信”、做到“两个维护”。认真落实中央八项规定及实施细则精神，驰而不息整治“四风”，力戒形式主义、官僚主义。发挥审计监督作用，抓好国资国企、重大项目、专项资金等重点领域及关键岗位的廉政风险防控。

　　各位代表，使命呼唤担当，实干成就梦想。我们将在市委的坚强领导下，在市人大、市政协的监督支持下，坚定信心、闻鸡起舞、日夜兼程、风雨无阻、狠抓落实，确保圆满完成今年的各项目标任务，以优异成绩庆祝中华人民共和国成立70周年！
